# Food insecurity, health care utilization, and health care expenditures

**DOI:** 10.1111/1475-6773.13283

**Published:** 2020-03-18

**Authors:** Emma Boswell Dean, Michael T. French, Karoline Mortensen

**Affiliations:** ^1^ Department of Health Management and Policy University of Miami Herbert Business School Coral Gables Florida

**Keywords:** access/demand/utilization of services, determinants of health/population health/socioeconomic causes of health, health care costs, health economics, social determinants of health

## Abstract

**Objective:**

To disentangle the relationships among food insecurity, health care utilization, and health care expenditures.

**Data Sources/Study Setting:**

We use national data on 13 465 adults (age ≥ 18) from the 2016 Medical Expenditure Panel Survey (MEPS), the first year of the food insecurity measures.

**Study Design:**

We employ two‐stage empirical models (probit for any health care use/expenditure, ordinary least squares, and generalized linear models for amount of utilization/expenditure), controlling for demographics, health insurance, poverty status, chronic conditions, and other predictors.

**Principal Findings:**

Our results show that the likelihood of any health care expenditure (total, inpatient, emergency department, outpatient, and pharmaceutical) is higher for marginal, low, and very low food secure individuals. Relative to food secure households, very low food secure households are 5.1 percentage points (*P* < .001) more likely to have any health care expenditure, and have total health care expenditures that are 24.8 percent higher (*P* = .011). However, once we include chronic conditions in the models (ie, high blood pressure, heart disease, stroke, emphysema, high cholesterol, cancer, diabetes, arthritis, and asthma), these underlying health conditions mitigate the differences in expenditures by food insecurity status (only the likelihood of any having any health care expenditure for very low food secure households remains statistically significant).

**Conclusions:**

Policy makers and government agencies are focused on addressing deficiencies in social determinants of health and the resulting impacts on health status and health care utilization. Our results indicate that chronic conditions are strongly associated with food insecurity and higher health care spending. Efforts to alleviate food insecurity should consider the dual burden of chronic conditions. Finally, future research can address specific mechanisms underlying the relationships between food security, health, and health care.

**What This Study Adds**

- The over 40 million individuals in the United States who experience food insecurity tend to have greater health care utilization and significantly higher annual health care expenditures relative to individuals who are not food insecure.
- Employing a more nuanced categorization of food security (very low, low, marginal, and secure) and analyzing survey data with contemporaneous measures of food security, our study builds off the existing literature to examine associations among food insecurity, health care utilization, and corresponding expenditures, while incorporating a variety of outcomes, key predictors including chronic conditions, and control variables.
- Our results generally align with previous literature in that we find a statistically significant increase in the likelihood of any health care utilization as well as conditional (on any utilization) health care expenditures as household food insecurity increases.
- When we incorporate controls for chronic health conditions, these differences across food security groups largely dissipate, suggesting that underlying heterogeneity in health status (proxied by the presence of chronic health conditions) is a key determining factor in driving these associations.

## 1 BACKGROUND

Food insecurity, an important social determinant of health, occurs when access to adequate food is limited by income.^1^ Food insecurity is one of the most pressing challenges in society today. It impacts the health status and well‐being of over 15 million households, comprising over 40 million individuals in the United States (12 percent of the population).^2^, ^3^, ^4^ Of these food insecure households, nearly 40 percent, or 5.8 million households, were considered very low food secure in 2017, meaning they had difficulty at any point during the year providing enough food for all family members.^2^

A growing literature demonstrates that food insecurity is a strong predictor of poor physical and mental health, as well as preventable chronic conditions among adults^5^, ^6^, ^7^, ^8^, ^9^, ^10^, ^11^, ^12^, ^13^, ^14^ and the elderly.^15^, ^16^, ^17^, ^18^, ^19^ Food insecurity can affect health through a number of pathways, including a less healthy diet than necessary to sustain health, fluctuations in dietary intake that renders management of chronic disease a challenge, and the prioritization of food purchases over health care needs such as prescription refills or preventive care.^10^

Those who experience food insecurity tend to have greater health care utilization and significantly higher annual health care expenditures relative to individuals who are not food insecure.^7^, ^20^, ^21^, ^22^ Research has shown that the increase in expenditures is most pronounced for people with chronic conditions such as diabetes, hypertension, and heart disease.^7^, ^11^ The odds of becoming a high‐cost health care user in the future are 46 percent greater for food insecure individuals than for those who are food secure.^23^ Despite these statistics, few physician practices (29.6 percent) and hospitals (39.8 percent) screen for food insecurity.^24^

The relationships among food insecurity, poor health (as reflected by the presence of chronic disease(s)), and health care expenditures are complicated to disentangle.^1^, ^8^, ^25^ For example, data available to researchers do not allow for causal inferences as to whether poor health led to higher health care spending and thus food insecurity (ie, reverse causality), or whether food insecurity led to poor health and thus higher health care expenses.^1^, ^10^, ^25^ Chronic health conditions are positively associated with health care utilization and spending, and food insecure individuals are more likely to exhibit chronic conditions.^26^, ^27^, ^28^ Individuals in food insecure households have more than two times the risk of developing type 2 diabetes relative to those in food secure households.^29^ Almost 80 percent of the growth in health care spending nationwide is attributable to the treatment of chronic diseases.^30^ Rising treatment costs for individuals with chronic health conditions impose a significant financial burden on individuals, which may cause them to forgo spending on necessities such as food, heat, or proper shelter.^26^ Thus, investigating the relationships between health care utilization/spending and food insecurity must account for the dynamics of chronic conditions.

Our study builds off the existing literature to examine associations among food insecurity, health care utilization, and corresponding expenditures, while incorporating a variety of outcomes, key predictors including chronic conditions, and control variables. We improve upon the literature by: (a) employing recently collected data that include relevant outcome and explanatory variables captured in the same time period and dataset, (b) specifying a more detailed and well‐validated definition of food security that aligns with the US Department of Agriculture's (USDA's) criteria (ie, high, marginal, low, very low), similar to that used with Canadian data,^5^ and (c) controlling for the presence of nine chronic conditions. Findings from this study have important policy implications for individuals, health care institutions, and policy makers who are trying to understand the complex relationships between household‐specific food insecurity, health status, and health care utilization/spending.

## 2 METHODS

### 2.1 Data source

We analyze recent data from the 2016 Medical Expenditure Panel Survey (MEPS), the first year food security measures are captured in this dataset. MEPS is a longitudinal survey, which is administered by the Agency for Healthcare Research and Quality to obtain information on health care utilization, expenditures, sources of payment, and insurance coverage for individuals residing in the United States.^31^

Our sample is derived from individuals participating in the MEPS Household Survey for whom food security data were collected in 2016. A total of 34 655 individuals comprise the full MEPS Household dataset. Food insecurity measures are asked of the reference person of record for each family unit. As MEPS only uses adults as the reference person of record for a family unit, our sample includes adults ages 18 and older. Filtering based on the reference person for each household brings the total number of observations to 13 877. Of these, 394 did not participate in the food insecurity survey and 17 did not fully answer the questions required to classify level of food insecurity and thus are excluded from the analysis. After these exclusions, our final analysis sample includes full records on 13 465 adults.

### 2.2 Measures

Our key predictor variable is a household's level of food insecurity. Rather than following the conventional approach in the existing literature of classifying households dichotomously as “food secure” or “food insecure,” we create a more disaggregated classification to align closely with the USDA 10‐item Adult Food Security Scale for food insecurity.^5^, ^32^, ^33^ Households are designated as having high, marginal, low, and very low levels of food security based on a battery of questions.

The USDA methodology adds one point for each affirmative response to the 10‐item Adult Food Security scale. Households defined as having high food security had no reported indications of food‐access limitations or issues (raw score = 0). Those with marginal food security had no indication of recent changes in food intake due to affordability issues, but reported anxiety over food sufficiency or a lack of food in the household (raw score = 1‐2). Low food security households had high levels of anxiety over food sufficiency and lack of food in the household, or limited food intake due to affordability issues (raw score = 3‐5). Lastly, households defined as having very low food security report both reduced food intake and disrupted eating as well as high levels of anxiety over food sufficiency and lack of food in the household (raw score = 6‐10).

The food insecurity variable described above is created by summing positive answers to the following questions (please see the flowchart in the Appendix S1 for the mapping of the MEPS food security questions into the four food security categories):

1. “How often in the last 30 days has anyone in the household worried whether food would run out before getting money to buy more?”
2. “How often in the last 30 days did the food purchased not last and the person/household didn't have money to get more?”
3. “How often in the last 30 days could the person/household not afford to eat balanced meals?”
  If any of the above three questions were answered affirmatively, the next several questions were asked:
4. “In the last 30 days, did the person/household reduce or skip meals because there wasn't enough money for food?”
5. “How many meals were skipped in the last 30 days?”
6. “In the last 30 days, did the person/household ever eat less because there wasn't enough money for food?”
7. “In the last 30 days, was the person/household ever hungry but didn't eat because there wasn't enough money for food?”
8. “In the last 30 days, did anyone in the household lose weight because there wasn't enough money for food?”
  And if any of the above questions were answered positively, the following questions were assessed:
9. “In the last 30 days, did anyone in the household not eat for a whole day because there wasn't enough money for food?”
10. “How many days in the last 30 days did anyone in the household not eat for a whole day because there wasn't enough money for food?”

The USDA questions pertain to the past 12 months, whereas MEPS uses a 30‐day window. To foster comparability, we follow the USDA Economic Research Service guidelines and adjust the 30‐day window in MEPS to align more closely with the 12‐month window in USDA.^33^ To do so, we increased the raw score by one if the responses to Questions 5 and 10 were three or more meals/days. Food insecurity is associated with other risk factors that may lead to changes in health care utilization and expenditures. We are able to adjust partially for these risk factors through a number of control variables in our statistical analysis. Although our set of control variables is extensive, we cannot rule out the possibility of important explanatory variables being omitted. Thus, our estimates, like those of others in the literature, should not be viewed as causal effects. The control variables include age, gender, race/ethnicity (Hispanic, non‐Hispanic white, non‐Hispanic black, non‐Hispanic Asian, non‐Hispanic other), educational attainment (less than high school, high school or GED, Bachelor's degree, Graduate degree, or other degree), marital status (married, single, separated or widowed, or divorced), employment status (employed, not employed), region of residence (South, Northeast, Midwest, West), health insurance status (Private, Medicaid, Medicare, other public insurance, uninsured, over 65, and no Medicare), and income (high‐income, middle‐income, low‐income, near poor, poor). In some analyses, we also control for a common set of chronic health conditions as defined in the MEPS survey. These chronic conditions are high blood pressure, heart disease, high cholesterol, diabetes, stroke, emphysema, cancer, arthritis, and asthma.

We analyze a number of outcome variables that cover different components of health care utilization and spending. Annual utilization measures include outpatient department visits, inpatient hospital discharges, emergency department (ED) visits, and prescription medication fills as a measure of pharmaceutical utilization. The latter outcome is a count of all prescribed medicines purchased by the respondent in 2016, including initial purchases and refills, not adjusted for number of days supplied per fill. Additionally, we analyze a number of annual health care spending outcomes, including total health care expenditures, outpatient expenditures, inpatient expenditures, ED expenditures, and pharmaceutical expenditures. We also examine medical care charges, including total charges, outpatient charges, inpatient charges, and emergency room charges. Given that results for medical care charges mirrored those of medical care expenditures, we do not formally present charge results in the study, but these analyses are available from the authors on request.

### 2.3 Statistical analysis

We first conduct a set of descriptive analyses, computing weighted mean values for each of our control variables separately by level of household food insecurity. We estimate chi‐squared statistics with second‐order Rao and Scott corrections for categorical variables, and Kruskal‐Wallis tests for noncategorical data (mean number of chronic conditions and number of months on Supplemental Nutrition Assistance Program (SNAP).^34^ Next, we compute and plot the weighted means and standard deviations for each of our outcome variables, overall and separately by level of household food insecurity. As these outcome measures are highly skewed, we also compute percentage of the relevant population with no health care expenditures as well as percentage of the relevant population with “high” health care expenditures (defined as approximately the top five percent of overall health care spending). Lastly, we calculate average health care utilization, overall and by level of household food insecurity. All descriptive analyses are adjusted for MEPS sampling weights to address the complex survey design.

Our main empirical specification comprises a two‐part model, with probit estimation in the first stage to indicate whether a person had any health care utilization or expenditures. Because probit applies maximum likelihood estimation (MLE) to nonlinear models, we estimate and present marginal effects averaged over the sample, with relevant standard errors calculated using the delta method. The second stage outcome is the natural log of medical care utilization or expenditures (conditional on having any utilization or expenditures), estimated using ordinary least squares (OLS). All models include level of household food insecurity, a rich set of control variables, and a series of chronic health conditions as outlined above. As with the descriptive analyses, all multivariate analyses adjust for MEPS sampling weights to account for the complex survey design.

We also employ an alternative estimation approach with a probit model in the first stage and a generalized linear model (GLM) with a log link and gamma family in the second stage. This alternative approach provides results that are very similar in direction, magnitude, and statistical significance to the main results. The full set of findings from these alternative specifications can be provided by the authors on request.

## 3 RESULTS

Table 1 presents weighted mean values for each of the explanatory variables used in our multivariate analyses, calculated overall and by level of household food security. The table also includes p‐values associated with chi‐squared and Kruskal‐Wallis tests to assess whether statistically significant differences are present in mean values for food secure and insecure households. Survey respondents from food insecure households are significantly less likely to be elderly (ie, age 65 and older), more likely to be female, more likely to be Black or Hispanic, and more likely to have lower levels of educational attainment. Additionally, food insecure households are more likely to come from low‐income households, more likely to have Medicaid insurance or be uninsured, less likely to have private insurance or Medicare, less likely to be married, less likely to be employed, more likely to have at least one chronic health condition, and more likely to receive SNAP.

**Table 1:** Descriptive statistics for all analysis variables, by level of food security

	Household food security					*P* value
	Total	Secure	Marginal	Low	Very low	
Age						
18‐24	5.56%	5.03%	8.59%	8.22%	7.09%	<.001
25‐34	18.47%	17.65%	24.23%	23.77%	19.38%	
35‐44	16.52%	16.17%	18.39%	19.62%	17.22%	
45‐54	17.86%	17.62%	16.16%	19.82%	22.29%	
55‐64	18.13%	18.26%	15.69%	16.81%	20.47%	
65‐74	13.47%	14.20%	10.64%	7.67%	11.24%	
75 and older	9.99%	11.08%	6.29%	4.08%	2.30%	
Gender						
Male	47.23%	48.85%	41.41%	38.58%	37.65%	<.001
Female	52.77%	51.15%	58.59%	61.42%	62.35%	
Race						
White	78.15%	79.78%	70.35%	69.39%	70.81%	<.001
Black	12.86%	11.16%	20.72%	22.39%	20.56%	
Other (combined category)	8.99%	9.06%	8.93%	8.22%	8.63%	
Ethnicity						
Hispanic	13.67%	11.97%	22.42%	29.52%	14.81%	<.001
Non‐Hispanic	86.33%	88.03%	77.58%	70.48%	85.19%	
Highest level of education						
Less than High School	10.18%	8.20%	17.96%	22.42%	19.85%	<.001
High School Diploma/Equivalent	46.09%	43.93%	56.34%	57.65%	56.62%	
Bachelor's Degree	21.56%	24.00%	11.09%	8.33%	8.87%	
Graduate Degree	12.10%	12.41%	11.02%	8.17%	12.27%	
Income as % of federal poverty line						
Poor (<100% FPL)	13.51%	9.73%	29.39%	29.68%	37.96%	<.001
Near poor (100% to <125% FPL)	4.29%	3.53%	5.57%	10.05%	9.34%	
Low Income (125% to <200% FPL)	13.02%	11.47%	18.44%	21.56%	22.69%	
Middle Income (200% to <400% FPL)	28.92%	28.89%	31.20%	32.09%	23.49%	
High Income (>=400% FPL)	40.27%	46.38%	15.40%	6.61%	6.53%	
Health insurance						
Private insurance	56.80%	60.10%	45.99%	40.21%	33.80%	<.001
Medicaid	10.73%	7.39%	22.11%	29.74%	31.39%	
Other public	1.49%	1.05%	2.45%	3.46%	5.51%	
Uninsured	7.55%	6.20%	12.74%	14.88%	15.75%	
Medicare (over 65)	23.16%	24.96%	16.45%	11.66%	13.54%	
Uninsured (over 65)	0.27%	0.30%	0.27%	0.06%	0.00%	
Marital status						
Married	47.14%	50.64%	33.91%	31.81%	23.45%	<.001
Single	25.33%	23.36%	33.44%	35.10%	37.08%	
Divorced/Widowed/Separated	27.53%	26.00%	32.65%	33.09%	39.47%	
Employment status						
Employed	67.39%	68.88%	64.77%	62.66%	51.79%	<.001
Unemployed	32.61%	31.12%	35.23%	37.34%	48.21%	
Region						
South	37.76%	37.35%	37.76%	40.30%	41.47%	.037
Northeast	17.55%	18.24%	15.32%	13.55%	13.36%	
Midwest	21.45%	21.21%	24.70%	21.18%	21.27%	
West	23.24%	23.20%	22.22%	24.97%	23.90%	
Chronic conditions						
Any conditions	65.57%	65.14%	62.09%	64.91%	77.27%	<.001
Mean conditions	1.63	1.60	1.57	1.72	2.18	<.001
Supplemental Nutrition Assistance Program (SNAP)						
Receive SNAP	10.75%	6.74%	23.46%	32.06%	38.63%	<.001
Number of months on SNAP	1.10	0.70	2.36	3.22	3.82	<.001
N	13 465	10 415	1 237	949	864	
% of Total (unweighted)	100.00	77.35	9.19	7.05	6.42	
% of Total (weighted)	100.00	83.10	7.01	4.69	5.20	

**Figure 1:**
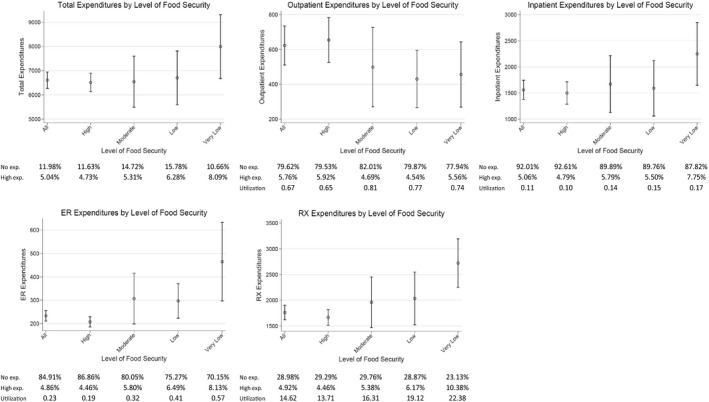
Medical care expenditures by level of food security

Figure 1 displays weighted mean value plots and 95% confidence intervals for all outcome variables, by level of household food security. Comparing groups shows that unadjusted total annual health care expenditures are larger for households with very low levels of food security: $6511 for high food secure households, $6581 for marginally food secure households, $6728 for low food secure households, and $7972 for very low food secure households (*P* < .01). This is driven partially by a larger percentage of households with high health care spending. While average outpatient expenditures are highest for households with high food security, average inpatient expenditures mirror total health care spending, with very low food secure households having higher average inpatient expenditures, driven both by a higher percentage of households having any inpatient spending and more households having high levels of total inpatient spending. Spending trends are even more transparent for ED and pharmaceutical expenditures, which successively rise as household food insecurity increases.

The statistics presented in Figure 1 provide aggregated and unadjusted information on how food insecurity is correlated with health care utilization and spending, but it is unclear whether food insecurity itself is the key driver of these relationships or some other underlying factors associated with food insecurity that have a bigger influence. As noted earlier, we control for many of these underlying factors in our multivariate models. Estimation results from our two‐stage specifications are shown in the top and bottom panels of Tables 2 and 3. Columns labeled probit represent any health care usage or expenditure, while columns labeled OLS represent the conditional health care usage or expenditure. Both the first and second stages include our main set of control variables, with the bottom panel adding control variables for the set of chronic health conditions outlined in the previous section.

**Table 2:** Selected estimation results for household food security and medical care utilization

Food security	Two‐part model—social and economic controls							
	(1)	(2)	(3)	(4)	(5)	(6)	(7)	(8)
	Outpatient utilization		Inpatient utilization		ED utilization		Pharma utilization	
	Probit	OLS	Probit	OLS	Probit	OLS	Probit	OLS
Baseline is food secure								
Marginal								
Estimate	0.002	0.052	0.015	−0.035	0.041**	0.007	0.055**	0.096
SE	0.015	0.097	0.010	0.047	0.015	0.038	0.015	0.056
Low								
Estimate	0.042	0.074	0.013	−0.008	0.092***	−0.020	0.067***	0.276***
SE	0.020	0.091	0.011	0.056	0.021	0.038	0.015	0.066
Very low								
Estimate	0.037	−0.006	0.023	0.031	0.112***	0.119**	0.104***	0.275***
SE	0.020	0.092	0.012	0.038	0.019	0.045	0.016	0.066

Food security	Two‐part model—social, economic, and chronic health condition controls							
	OP utilization		IP utilization		ED utilization		Pharma utilization	
	Probit	OLS	Probit	OLS	Probit	OLS	Probit	OLS
Baseline is food secure								
Marginal								
Estimate	−0.004	0.030	0.008	−0.040	0.031*	0.002	0.039**	0.032
SE	0.015	0.092	0.010	0.046	0.014	0.037	0.014	0.052
Low								
Estimate	0.023	0.049	0.001	−0.027	0.073***	−0.032	0.035	0.171**
SE	0.018	0.091	0.010	0.055	0.017	0.039	0.018	0.058
Very low								
Estimate	0.001	−0.047	0.003	0.016	0.078***	0.101*	0.051**	0.088
SE	0.019	0.090	0.011	0.041	0.018	0.045	0.019	0.063

Note

Social and Economic Controls include the following: age, gender, race, ethnicity, education, income, health insurance, marital status, employment status, region, and SNAP receipt. Chronic Health Conditions Controls include the following: high blood pressure, heart disease, high cholesterol, diabetes, cancer, arthritis, and asthma. Probit estimates are presented as marginal effects, averaged over the sample. Statistical significance at the 5%, 1%, and 0.1% levels are noted as *, **, and ***, respectively.

**Table 3:** Estimation results for household food security and medical care expenditures

Food security	Two‐part model—social and economic controls									
	(1)	(2)	(3)	(4)	(5)	(6)	(7)	(8)	(9)	(10)
	All expenditures		OP expenditures		IP expenditures		ED expenditures		Pharma expenditures	
	Probit	OLS	Probit	OLS	Probit	OLS	Probit	OLS	Probit	OLS
Baseline is food secure										
Marginal										
Estimate	0.026***	0.132*	0.002	0.133	0.015	−0.145	0.036*	−0.126	0.054***	0.186*
SE	0.010	0.065	0.015	0.142	0.010	0.147	0.015	0.145	0.015	0.093
Low										
Estimate	0.026**	0.252**	0.041*	−0.127	0.013	−0.033	0.084***	−0.156	0.067***	0.369***
SE	0.011	0.083	0.020	0.161	0.011	0.151	0.018	0.113	0.016	0.101
Very low										
Estimate	0.051***	0.248*	0.036	−0.043	0.022	0.007	0.100***	0.081	0.101***	0.517***
SE	0.011	0.097	0.019	0.16	0.013	0.168	0.020	0.107	0.016	0.111

Food security	Two‐part model—social, economic, and chronic health condition controls									
	All expenditures		OP expenditures		IP expenditures		ED expenditures		Pharma expenditures	
	Probit	OLS	Probit	OLS	Probit	OLS	Probit	OLS	Probit	OLS
Baseline is food secure										
Marginal										
Estimate	0.017	0.053	−0.004	0.096	0.008	−0.18	0.027	−0.145	0.038**	0.088
SE	0.010	0.063	0.015	0.139	0.009	0.147	0.014	0.145	0.014	0.089
Low										
Estimate	0.011	0.093	0.023	−0.185	0.001	−0.054	0.065***	−0.176	0.035	0.201*
SE	0.012	0.077	0.018	0.172	0.010	0.15	0.017	0.115	0.018	0.091
Very low										
Estimate	0.026*	−0.013	0.009	−0.134	0.002	0.021	0.069***	0.049	0.047*	0.213
SE	0.013	0.097	0.019	0.155	0.011	0.165	0.019	0.111	0.019	0.113

Note

Social and Economic Controls include the following: age, gender, race, ethnicity, education, income, health insurance, marital status, employment status, region, and SNAP receipt. Chronic Health Conditions Controls include the following: high blood pressure, heart disease, high cholesterol, diabetes, cancer, arthritis, and asthma. Probit estimates are presented as marginal effects, averaged over the sample. Statistical significance at the 5%, 1%, and 0.1% levels are noted as *, **, and ***, respectively.

Table 2 presents regression results for medical care utilization, focusing separately on outpatient, inpatient, ED, and pharmaceutical utilization. The first panel of Table 2, which includes social and economic control variables, shows that food insecurity is significantly and positively associated with having any ED visit (*P* = .007 for marginal food security, *P* < .001 for low food security, *P* < .001 for very low food security) or pharmaceutical utilization (*P* < .001 for all three levels of food insecurity), as well as with number of ED visits for very low food secure households (*P* = .008) and with total pharmaceutical utilization (*P* = .091 for marginal food security, *P* < .001 for low food security, *P* < .001 for very low food security). The second panel of Table 2 adds in controls for a broad set of chronic conditions and confirms these relationships, although with smaller coefficient estimates and slightly larger p‐values.

Table 3 presents regression results for medical care expenditures, focusing on total, outpatient, inpatient, ED, and pharmaceutical spending. The first panel of Table 3, which includes social and economic controls, but not health indicators, clearly shows that level of household food insecurity is positively associated with having any health care expenditures, as well as total expenditures conditional upon having any utilization. Households with marginal, low, and very low food security are increasingly more likely than food secure households to have any health care expenditures (*P* = .006 for marginal food security, *P* = .021 for low food security, *P* < .001 very low food security), and have higher levels of total health care expenditures (*P* = .044 for marginal food security, *P* = .003 for low food security, *P* = .011 for very low food security). These differences are not driven exclusively by inpatient or outpatient expenditures, however, as outpatient and inpatient spending are rarely significantly different across households with different levels of food insecurity. The exception is very low food secure households, which have a higher likelihood of any outpatient expenses (*P* = .041) compared to food secure households. Large quantitative differences are seen for ED and pharmaceutical expenditures. As household food insecurity increases, the likelihood of any ED expenditure rises significantly, though ED spending conditional on any usage is not significantly different across groups. Any pharmaceutical spending (*P* < .001 for all three levels of food insecurity) along with conditional expenditures (*P* = .047 for marginal food security, *P* < .001 for low food security, *P* < .001 for very low food security) rises significantly as household food insecurity increases.

As expected, underlying chronic health conditions are positively associated with health care utilization and spending.^26^, ^27^, ^28^ In the second panel of Table 3, we add in a set of variables for chronic health conditions to see how this impacts our earlier regression results. With these controls now in the models, the significant differences in total health care spending across the food security groups largely disappear. The likelihood of any and conditional health care expenditures is now statistically similar across levels of household food security. The only exception is for low food secure households, who are slightly more likely to have any health care expenditures (*P* = .045). However, controlling for chronic health conditions does not mitigate the statistically significant differences in the likelihood of any ED spending. Again, as household food insecurity increases, the likelihood of any ED expenditure rises significantly (*P* = .057 for marginal food security, *P* < .001 for low food security, *P* < .001 for very low food security), although total ED spending conditional on any usage is not significantly different for households with different levels of food insecurity. The link between food insecurity and pharmaceutical expenditures weakens somewhat when controlling for chronic health conditions, but some significant differences remain—households across all levels of food insecurity are more likely to have any pharmaceutical expenditures (*P* = .007 for marginal food security, *P* = .055 for low food security, *P* = .013 for very low food security) though only low food secure households have significant differences in conditional spending (*P* = .029).

To better understand the connections between chronic health conditions and food insecurity, we ran two‐stage models separately by type of chronic health condition. These regressions include our standard social and economic controls, in addition to a binary control variable indicating whether a respondent has multiple chronic conditions. The sample sizes for respondents with emphysema and stroke were not sufficient to run our full set of specifications, and thus, we do not report results for respondents with these two chronic conditions. Results (see Table 4) show a significant link between any health care spending and food insecurity among heart disease patients, but conditional health care spending for heart disease patients was no different for food secure and insecure households. For other chronic health conditions, health care expenditures are generally similar for food secure and food insecure households.

**Table 4:** Estimation results for household food security and medical care expenditures, by chronic condition

Food security	Two‐part model—social and economic controls													
	(1)	(2)	(3)	(4)	(5)	(6)	(7)	(8)	(9)	(10)	(11)	(12)	(13)	(14)
	High blood pressure		Heart disease		High cholesterol		Diabetes		Cancer		Arthritis		Asthma	
	Probit	OLS	Probit	OLS	Probit	OLS	Probit	OLS	Probit	OLS	Probit	OLS	Probit	OLS
Baseline is food secure														
Marginal														
Estimate	0.016*	0.128	0.019*	0.129	−0.004	0.002	0.01	0.104	0.007	0.102	0.018**	0.112	−0.021	−0.226
SE	0.007	0.092	0.009	0.144	0.009	0.104	0.007	0.134	0.013	0.168	0.006	0.104	0.021	0.183
Low														
Estimate	0.002	0.110	0.023**	0.014	−0.009	0.072	−0.011	0.199	0.018*	−0.025	−0.005	0.262*	−0.927	−0.080
SE	0.012	0.109	0.009	0.113	0.013	0.102	0.020	0.129	0.010	0.204	0.017	0.104	−0.041	0.145
Very low														
Estimate	0.009	0.011	0.019**	−0.044	0.001	−0.045	0.005	0.106	−0.008	0.325	0.005	−0.075	−0.001	−0.076
SE	0.011	0.117	−0.009	0.175	0.011	0.097	0.011	0.137	0.017	0.175	0.008	0.119	0.022	0.186
N	4838		1949		4341		1218		1260		3727		1221	

Note

Social and Economic Controls include the following: age, gender, race, ethnicity, education, income, health insurance, marital status, employment status, region, and SNAP receipt. Probit estimates are presented as marginal effects, averaged over the sample. Statistical significance at the 5%, 1%, and 0.1% levels are noted as *, **, and ***, respectively.

## 4 DISCUSSION

A growing body of literature establishes a strong association between food insecurity, greater health care utilization, and higher medical care spending. We add to this literature by employing a more nuanced categorization of food security and analyzing newly available survey data—with contemporaneous food security measures—which allows us to control for a broad set of economic, social, and health‐related factors. Our results generally align with previous literature in that we find a statistically significant increase in the likelihood of any health care utilization as well as conditional (on any utilization) health care expenditures as household food insecurity increases. However, when we incorporate controls for chronic health conditions, these differences across food security groups largely dissipate, suggesting that underlying heterogeneity in health status (proxied by the presence of chronic health conditions) is a key determining factor in driving these associations.

Our results differ in a number of meaningful ways from two recent studies. Berkowitz et al found a significant link between food security and health care expenditures for diabetes, hypertensive, and heart disease patients.^7^ Our results are directionally similar, but nonsignificant, particularly for conditional expenditures. When we replicate the methodology of Berkowitz et al^7^ with our data, the main results are largely unchanged, suggesting variability in controls or variable definitions is not driving the differences that we find. The second study, also by Berkowitz et al,^22^ found that food insecurity was associated with more ED visits and hospitalizations. In our analysis, we find that only the ED relationship—and to a lesser extent the pharmaceutical relationship—persists. The timeframe for each study might be a potential explanation for the differences. The Berkowitz et al studies assemble household food security measures from 2011 and health care expenditure data from 2012 and 2013. If the impacts of food insecurity on household spending compound over time, increased health care expenditures may emerge in the long‐term even though they are not observed in the short‐term. This could shed light on why their studies detect significant differences in inpatient spending while ours does not. In addition, Berkowitz and colleagues use a dichotomous definition of food security (secure versus insecure) that differs from our more nuanced categorical approach.

It is important to emphasize that our findings do not necessarily imply a direct causal link between food insecurity, health care utilization, and corresponding expenditures. Nevertheless, food insecurity could directly contribute to chronic health conditions—particularly for conditions such as high blood pressure, high cholesterol, diabetes, and heart disease. This might indirectly relate to a number of chronic health conditions through its impact on stress or anxiety. Food insecure households might also be more likely to have unobserved social and economic factors that lead to lower health care expenditures in the absence of food insecurity, such as lack of reliable transportation or irregular work schedules.

The relationships among chronic health conditions, health care utilization, and expenditures may impact a household's level of food security through a number of channels. If a household member is unable to work at his/her full capacity due to an underlying health condition, or if a household unit has to devote a significant portion of income to expenses associated with chronic health conditions, then the unit might have fewer resources to devote to a healthy, balanced diet. The stress and anxiety associated with an inability to provide an adequate quantity and quality of food for oneself and/or a household might also indirectly affect the management of chronic conditions. Chronic conditions can exert significant financial pressure on the household food budget. These pressures may be substantial enough to increase the risk of food insecurity. Thus, interventions to address food insecurity could be targeted to those with the highest need, as demonstrated by the presence of chronic health conditions.

A renewed focus is underway in the United States highlighting the importance of social determinants of health.^24^ The Centers for Medicare & Medicaid Services (CMS) announced in November 2018 that it is exploring the use of Medicaid funds for hospitals and health systems to directly pay for healthy food and basic shelter.^35^ To this point, we find that food insecurity is associated with both an increased likelihood of any use and conditional utilization of ED services and pharmaceuticals, even after controlling for underlying chronic health conditions. This result is especially poignant for patients, providers, and payers. In unreported analyses, we also found a strong link between food insecurity and ED usage among food insecure individuals with high blood pressure and high cholesterol. Thus, providing a healthy and reliable food source for food insecure individuals with these chronic health conditions could potentially lead to decreased ED utilization, lower associated expenditures, and possibly net overall health system savings.

Although we are unable to determine whether a true causal pathway is present between food insecurity and health care utilization/expenditures—food insecurity may correlate with a number of unobserved factors that also impact a person's health, access to care, and utilization—the associations reported here are timely and policy relevant. Indeed, we provide new information on what factors might be contributing to these associations, suggesting that underlying chronic health conditions are one channel that may be particularly important in understanding this link. To this end, several fruitful opportunities exist for future research. First, it would be informative to better understand the underlying mechanisms that drive the relationship between chronic health conditions and food insecurity. In particular do chronic health conditions cause food insecurity or vice versa. Second, health economists can calculate the net economic benefit of food assistance programs such as SNAP, TANF, and more fragmented local initiatives (eg, recycled food waste). Third, it would be interesting to study how food insecurity relates to some of the other social determinants of health (eg, obesity, substance use, risky health behaviors). Finally, health care professionals are uniquely positioned to simultaneously screen for chronic health conditions and food insecurity. Health services researchers can team with clinicians to develop screening tools and evaluate programs that provide food assistance to those who are most in need—food insecure individuals with comorbid chronic conditions.

## Supporting information

Author matrix

Appendix S1
