# Supplementary material for: Food insecurity, health care utilization, and health care expenditures
Source: Health Serv Res. 2020 Mar 18;55(Suppl 2):883–93. doi: 10.1111/1475-6773.13283 (PMC7518817; doi:10.1111/1475-6773.13283)
Supplement: Supplementary file 2 — Appendix S1 [file HESR-55-883-s002.docx]

Online Appendix: Mapping MEPS food security variables into four food security categories

**Sum of Affirmative Responses**
